# Does the expression of granzyme B participate in inflammation, fibrosis, and fertility of hydatid cysts?

**DOI:** 10.1007/s00436-023-08056-2

**Published:** 2023-12-11

**Authors:** Dina Sweed, Mohamed Mohamady, Marwa A. Gouda, Yahya Fayed, Sara A. Saied, Sara M. Abd Elhamed

**Affiliations:** 1 Pathology Department, National Liver Institute, Menoufia University, Shebin Elkom, Menoufia, Egypt; 2 Clinical and Molecular Parasitology Department, National Liver Institute, Shebin Elkom, Menoufia, Egypt; 3 Hepatopancreatobiliary Surgery Department, National Liver Institute, Menoufia University, Shebin Elkom, Menoufia, Egypt; 4 Clinical Pathology Department, National Liver Institute, Menoufia University, Shebin Elkom, Menoufia, Egypt

**Keywords:** CD4, CD8, Fertility, Granzyme B, Hydatid cyst, Immune response

## Abstract

*Echinococcus granulosus* (sensu lato), a cestode that is endemic in Egypt, causes cystic echinococcosis (CE), a significant but neglected zoonotic disease that is prevalent throughout the world. Infected hydatid cysts are classified as fertile or non-fertile based on the presence of protoscoleces; nevertheless, the mechanism of non-fertile CE cysts remains unknown. The study aimed to assess whether granzyme B (GrB) expression and CD4 + /CD8 + could be related to the induction of non-fertile CE cysts. A total of fifty-eight individuals diagnosed with visceral hydatid cysts were selected, and they were further divided according to cyst fertility into fertile and non-fertile. Immunohistochemistry for CD4, CD8, and GrB was done. According to the results, hydatid cysts are common in adults and have no gender preference. The same clinical and laboratory data were shared by patients with fertile and non-fertile cysts (*p* = 0.186). GrB expression was not impacted by the fibrous deposition inside the hydatid cyst wall (*p* = 0.85); however, GrB was significantly correlated with the inflammatory density (*p* = 0.005). GrB expression was also found to be significantly higher in non-fertile cysts (*p* = 0.04). GrB expression is positively correlated with CD4 and CD8 expression. In conclusion, the expression of GrB in hydatid cysts may exacerbate the inflammatory response and impede cyst fertility while not affecting the fibrous deposition in the cyst wall.

## Introduction

Cystic echinococcosis (CE) or hydatidosis is a widespread zoonotic parasitic infection caused by the flatworm *Echinococcus granulosus* (*E. granulosus*) (sensu lato) (Aziz et al. 2011). The World Health Organization (WHO) has identified echinococcosis as one of the seventeen neglected tropical diseases that will be the focus of disease control or eradication efforts by 2050. Numerous regions across the globe, including Egypt, exhibit the disease; however, the current prevalence in Egyptian has yet to be determined (Abdelbaset et al. 2021). The global impact of CE is significant, affecting a population exceeding one million individuals and incurring annual economic expenses in addition to livestock losses.

Canids and other canines are definitive hosts due to their ability to harbor adult tapeworms and excrete parasite eggs in their fecal matter. Intermediate hosts are susceptible to infection by ingesting eggs introduced through contaminated food or water sources. As a result, the larva that emerges from the egg undergoes development into a hydatid cyst within the liver and lungs of the intermediate host (Romig et al. 2015). The human population serves as an intermediary host after the accidental ingestion of eggs, which subsequently mature into hydatid cysts. This condition can result in severe morbidity and mortality (Bonelli et al. 2018; Santucciu et al. 2020).

A hydatid cyst is a single-chambered structure within the internal organs of the intermediate host, which consists of three distinct layers, namely the germinal and laminated layers of parasite origin and the adventitial layer of host origin, which serves to encapsulate the parasite. The adventitial layer comprises collagen fibers, epithelioid cells, eosinophils, and lymphocytes. The germinal layer initiates the formation of the laminated layer inside the host and subsequently generates protoscoleces to sustain its life cycle. Certain cysts cannot generate protoscoleces and are categorized as non-fertile cysts. The precise molecular mechanisms underlying cyst fertility remain uncertain. Nevertheless, the immune response of the host organism may exert a pivotal influence in this regard (Hidalgo et al. 2019).

A diverse range of antigens is present in the hydatid cyst fluid of *E. granulosus*. The glycan antigens of *E. granulosus* expressed in protoscoleces, hydatid cyst fluid, and the laminated layer exhibit an extraordinary level of antigenicity. The parasite can limit glycan antigens’ expression on the laminated layer’s surface, thereby reducing the host’s immune responses (Rafiei Sefiddashti et al. 2017). In protoscoleces and metacestodes, two primary antigens, tegumentary antigen and antigen B (AgB), have been demonstrated to have a vital role in host immune evasion and inhibition of chemotaxis. Interfering with the immune response to protoscoleces, on the other hand, could confer advantages during the initial phases of the infection course but may elicit harmful effects during the subsequent stages (Ali 2022).

The fundamental mechanisms governing fertility are complex and may be linked to immunological response in addition to host or parasite variables. Understanding the local immune response may help establish new therapies and block immune evasion mechanisms (Ali 2022). Research studies have indicated that mediated T-cell responses are essential in the immunology of hydatid cysts, both in experimental mouse models and in patients with CE. *E. granulosus* can circumvent the host’s immune system by eliciting T-helper 1 (Th1) and T-helper 2 (Th2) responses. The modulation of CD4 + /CD8 + balance is crucial in developing hydatid cysts (Riganò et al. 2001) (Yasen et al. 2021). However, their role in cyst fertility needs to be better elucidated.

Granzyme B (GrB) is a serine protease enzyme that is synthesized and secreted by diverse cell types, including immune cells such as T and B lymphocytes, macrophages, and basophils (Velotti et al. 2020). The pro-apoptotic function of GrB is susceptible to modulation by various immune system cells, such as cytotoxic T lymphocytes and natural killer (NK) cells. GrB may have a promising role in the pathogenesis of various inflammatory conditions, such as acute and chronic inflammatory diseases as well as malignancies (Campos et al. 2020; Wang et al. 2021).

Herein, we aim to investigate the expression of CD4 + /CD8 + T cells and GrB in surgically resected hydatid cyst cases and to associate their expression with hydatid cyst fertility to clarify the immunological mechanisms underlying cyst fertility.

## Material and methods

### Ethical statement

The institutional review board scrutinized and endorsed the present study protocol at the National Liver Institute, Menoufia University, bearing reference number 00486/2023. Personal identifiers such as name and registration number were excluded during data compilation. Instead, a unique identifier number was assigned to each patient.

### Study design and population

This retrospective cohort study involved 58 formalin-fixed, paraffin-embedded specimens from patients diagnosed with visceral hydatid cysts between 2011 and 2020. As part of the clinical management of patients, specimens were procured from the archives of the Pathology Department. A positive serological test, imaging modalities, and histological results were used to establish the diagnosis. Age, gender, cyst location, cyst size, and focality were all reported along with the surgical management. Surgical treatment included endo-cystectomy in 87.9% and partial hepatectomy in 12.1% of cases.

### Exclusion criteria

Any patient who has received oral or intra-cystic injection therapy before surgery was excluded from the study.

### Histopathological study

The cases were divided into two groups based on the international consensus on echinococcosis terminology: fertile (defined by the germinal layer producing protoscoleces, which are then released into the parasite fluid) and non-fertile cyst (defined by the lack of viable protoscoleces). Non-fertile cysts can be further subdivided into viable non-fertile cysts with only detected germinal and laminated layers and non-fertile/non-viable cysts that are only made of granulation tissue, devoid of any live cells and, therefore, any parasitic structure (Hidalgo et al. 2019; Vuitton et al. 2020).

The adventitial layer was evaluated by assigning a score index to assess the extent of inflammation in the tissue. Two pathologists independently graded the inflammation, and they reached an agreement on the median count in a 10-high power field (HPF), which was subsequently scaled on a zero–three scale. Mild inflammation is characterized by the presence of up to 30 inflammatory cells per 10 HPF; moderate inflammation is indicated by the presence of 30 to 100 inflammatory cells, while severe (3) more than 100 inflammatory cells per 10 HPF. The pathologists assessed the inflammatory cells based on their morphology and staining pattern in hematoxylin and eosin (H&E) (Hidalgo et al. 2019).

Masson trichrome (MT) stain was used to determine the amount of collagen deposition in the wall of hydatid cysts. Slides were stained with Weigert’s iron hematoxylin working solution for 10 min, Biebrich scarlet-acid fuchsin solution for 15 min, phosphomolybdic–phosphotungstic acid solution for 15 min, and finally transferred to aniline blue solution for 10 min with adequate washing between each step, briefly rinsed with distilled water and incubated for 5 min in a solution of 1% acetic acid. The color of collagen fibers appeared blue, and thickness was assessed using a software package included in Olympus microscope CX41 using a DP26 camera.

### Serological testing

Serological test records were available for 32 patients. The serology for cystic echinococcosis diagnosis was performed with commercially available kits at the Clinical and Molecular Parasitology Department (ELI.H.A Echinococcus, REF 66604, ELITech MICROBIO, France). It was executed according to the guidelines provided by the manufacturer. The tests used in the study depended on the identification of antibodies directed against the crude hydatid fluid soluble antigen of *E. granulosus* species complex, except the immunoblot assay, which was designed to detect the crude hydatid fluid antigen of *Echinococcus multilocularis*. Titer < 1:80 was interpreted as a non-significant/negative reaction. From 1:80 ≤ T ≤ 1:160 was interpreted as a doubtful reaction, and a test with a titer ≥ 1:320 was considered a significant reaction in favor of hydatidosis progression.

### Immunohistochemical study

Tissue sections with a 4–5 µm thickness were obtained from every sample and deposited onto positively charged slides. The protocol involved the use of a ready-to-use mouse monoclonal CD8 antibody (Ref; N1592) and a ready-to-use mouse monoclonal CD4 antibody (Ref; IR649), both of which were obtained from DAKO, Texas, USA. Additionally, the GrB antibody was obtained from Santa Cruz Biotechnology, Texas, USA, and diluted to a 1:200 concentration (Cat. #sc-8022). The specimens were subjected to heat-mediated antigen retrieval for 20 min by immersing them in a high-pH Tris–EDTA solution (Dako, Ref K8000, Glostrup, Denmark). The sections were subjected to incubation with primary antibodies overnight at 4 °C. Negative control was incorporated into the experimental design.

### Antibodies assessment

The cases were evaluated based on positive/negative expression, whereby positive expression was defined as the presence of any inflammatory cells exhibiting positive cytoplasmic staining for CD4, CD8, and GrB antibodies. The percentage of CD4 + and CD8 + T cells, as well as GrB, was determined in five representative HPFs (× 400). Two independent pathologists evaluated the results and recorded them as the mean percent of positive cells (relative to the total cells)/HPF.

### Statistical analysis

The statistical assessment was performed utilizing the SPSS software version 26 (SPSS Inc., Chicago, Illinois, USA), commonly used in social sciences research. The Shapiro–Wilk test was performed on all data sets to ensure normal distribution. The results were expressed as mean ± standard error (SE). For quantitative variables, the significance of differences between groups was determined by one-way analysis of variance (ANOVA) followed by a post hoc Tukey test. Furthermore, the chi-square and Monte Carlo tests were used for qualitative data. The Pearson’s correlation coefficient was implemented to measure the statistical relationship between two continuous variables. The statistical significance of the two-tailed P-value was established to be less than or equal to 0.05.

## Results

### Clinicopathological data of the cases under study

The detailed clinicopathological data of the studied hydatid cyst cases are shown in Table 1.

**Table 1:** The clinicopathological data of studied cases

Variables	No. (%)
Age (years) (mean ± SE)	38.58 ± 13.43
Gender	
Male	27 (46.6%)
Female	31 (53.4%)
Serology (No = 32)	
Positive	18 (56.2%)
Negative	14 (43.8%)
Focality	
Single	40 (69%)
Multiple	18 (31%)
Cyst size (cm) (mean ± SE)	9.202 ± 4.761
Type of hydatid cyst	
Fertile	32 (55.2%)
Non-fertile/viable	21 (36.2%)
Non-fertile/non-viable	5 (8.6%)
Density of inflammation	
Mild	5 (8.6%)
Moderate	11 (19%)
Marked	42 (72.4%)
Fibrosis of the cyst wall (µm) (mean ± SE)	4618.448 ± 282.3

**Fig. 1:**
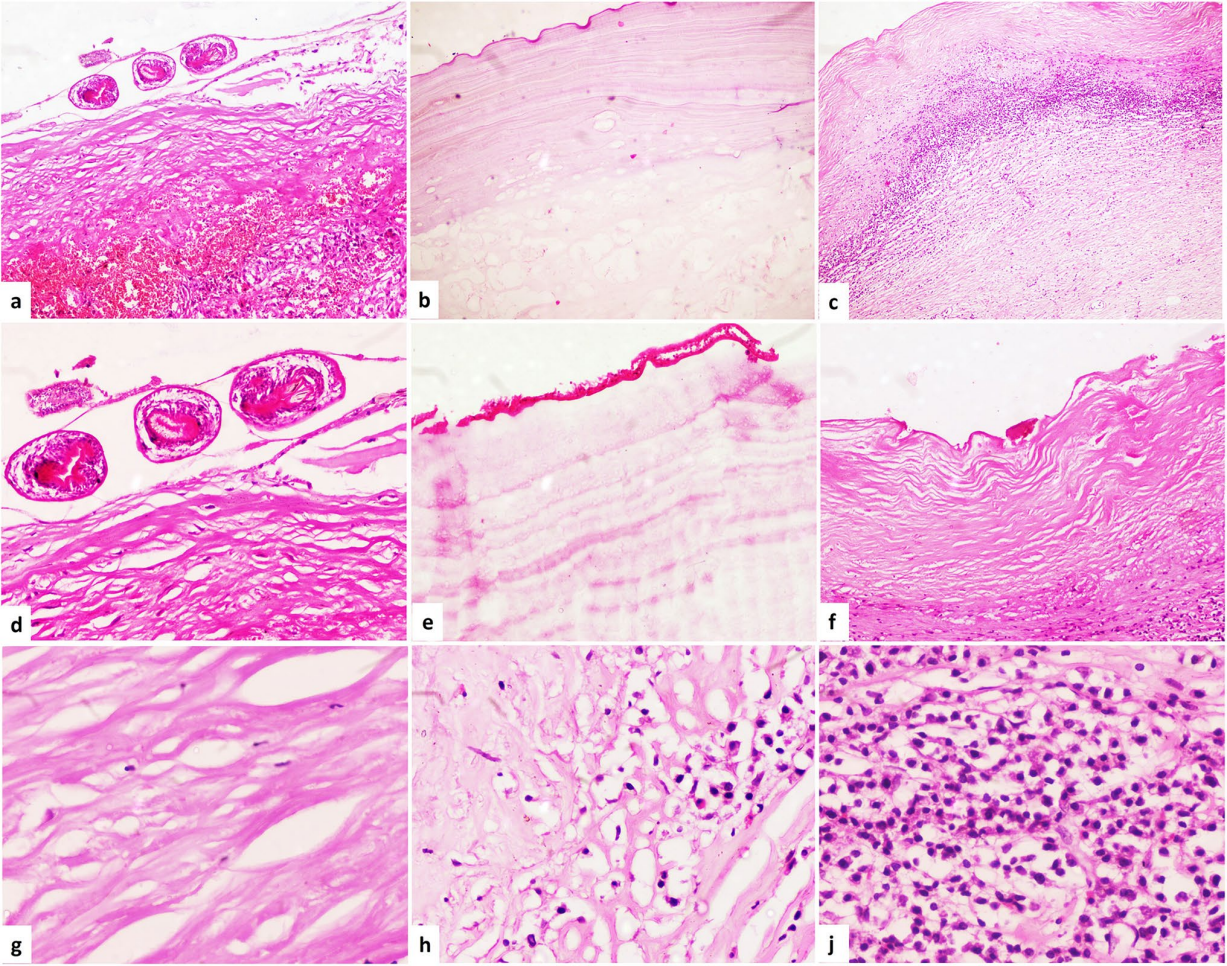
The histopathological features of fertile and non-fertile hydatid cysts. **a** Fertile hydatid cyst showing viable protoscoleces (PSC) and moderate inflammation at the adventitial wall of cyst (H&E, × 40). **b** Non-fertile hydatid cyst showing a thick laminated layer but lacking any PSC (H&E, × 40). **c**) Non-fertile/non-viable hydatid cyst showing an adventitial layer composed of marked inflammatory cellular infiltrate without PSC or laminated layer (H&E, × 40). **d** Fertile hydatid cyst showing viable protoscoleces (PSC) and refractile hooks (H&E, × 100). **e** Non-fertile hydatid cyst showing a germinal layer with underlying thick laminated layer but lacking any PSC (H&E, × 100). **f** Non-fertile/non-viable hydatid cysts showing a fibrosis and inflammatory cellular infiltrate without PSC or germinal layer (H&E, × 100). **g** Adventitial layer showed mild inflammation (< 30 inflammatory cells/10 HPF), (H&E, × 400). **h** Adventitial layer showed moderate inflammation (30–100 inflammatory cells/10 HPF) (H&E, × 400). **j** Adventitial layer showed marked inflammation (> 100 inflammatory cells/10 HPF), (H&E, × 400)

The average age at the presentation time was 38.58 years old, with nearly equal gender distribution. Positive serum for hydatid antibodies was found in 18 of 32 individuals, with reported tests accounting for 56.25% of the total. Serological investigations revealed positive results in ten fertile and eight non-fertile patients. Negative serum samples were observed in 14 (43.75% of the cases), with five of them fertile and the remainder non-fertile (viable in six cases or non-viable in three cases). Pathological evaluation revealed that one-third of the cases had multiple cyst formation with a mean cyst size of 9.2 cm. The cysts were divided into 55.2% fertile cases and 44.8% non-fertile cases, which are subdivided into 36.2% non-fertile/viable cysts and 8.6% non-fertile/non-viable (Fig. 1a–f).

Furthermore, we did not find both fertile and non-fertile cysts in the same patient. Multiple cysts within a patient were categorized as 11 fertile cysts, five non-fertile/viable cases, and three non-fertile/non-viable cases based on their fertility status. The density of the inflammatory infiltrate was mild in 8.6% of cases while moderate and marked in 19% and 72.4%, respectively (Fig. 1g–j).

There was no significant impact of the clinical, laboratory, and inflammatory density on the type of hydatid cyst (Table 2).

**Table 2:** Comparison between types of hydatid cysts regarding the clinical and laboratory data

Variables	Fertile	Non-fertile/viable	Non-fertile/non-viable	*p*-value test
Age (years) (mean ± SE)	36.2 ± 12.27	40.00 ± 14.48	49.0 ± 13.26	*p* = 0.168
Gender				
Male	15	10	2	*p* = 0.95
Female	17	11	3	
Serology (No = 32)				
Positive	10	8	0	*p* = 0.106
Negative	5	6	3	
Focality				
Single	21	16	3	*p* = 0.69
Multiple	11	5	2	
Cyst size (cm) (mean ± SE)	10.078 ± 5.256	8.667 ± 4.0	5.84 ± 2.61	*p* = 0.147
Density of inflammation				
Mild	4	1	0	*p* = 0.186
Moderate	7	3	1	
Marked	21	17	4	
Fibrosis (µm) (mean ± SE)	4315.8 ± 394.7	4766.5 ± 437.3	5933.6 ± 909.6	*p* = 0.27

**Fig. 2:**
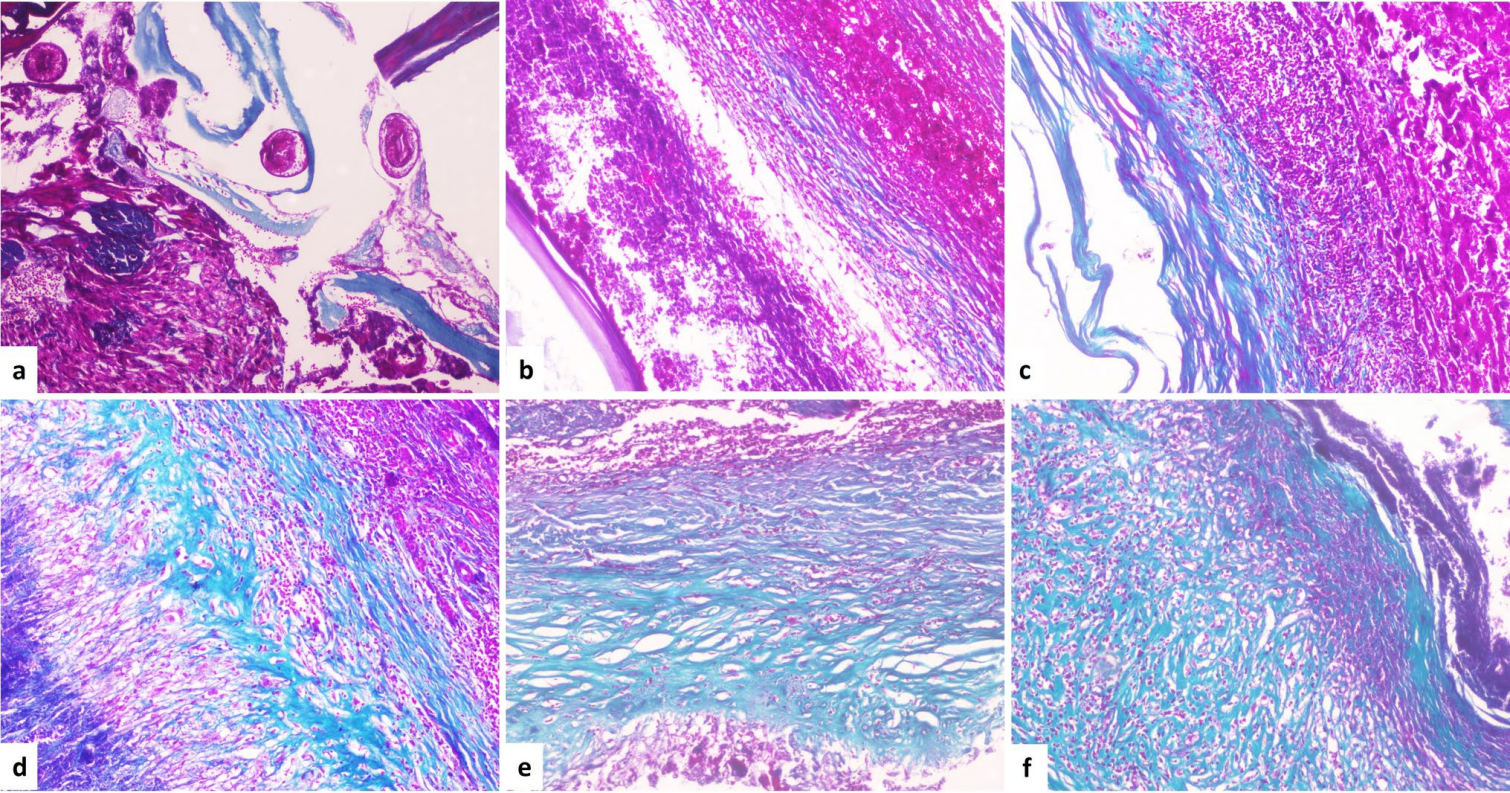
The distinct fibrous depositions within the cyst wall are emphasized by the Masson trichrome stain. **a** A fertile hydatid cyst showing focal fibrous deposition within the wall (MT, × 100), **b** Non-fertile/viable hydatid cyst showing mild fibrous deposition within the wall (MT, × 100). **c** Non-fertile/non-viable hydatid cyst showing moderate fibrous deposition within the wall (MT, × 100). **d** A hydatid cyst showing marked fibrous deposition within the wall interrupted by inflammatory cells (MT, × 100). **e** A hydatid cyst showing marked fibrous deposition within the wall (MT, × 100). **f** A hydatid cyst showing marked diffuse fibrous deposition within the wall (MT, × 100)

According to the data analysis, fibrous deposition within the cyst wall did not correlate with cyst fertility (*p* = 0.27) or inflammatory density (*p* = 0.66) (Fig. 2).

### Comparison between the fertile and non-fertile hydatid cysts regarding CD4 + , CD8 + T cells, and GrB expression

**Fig. 3:**
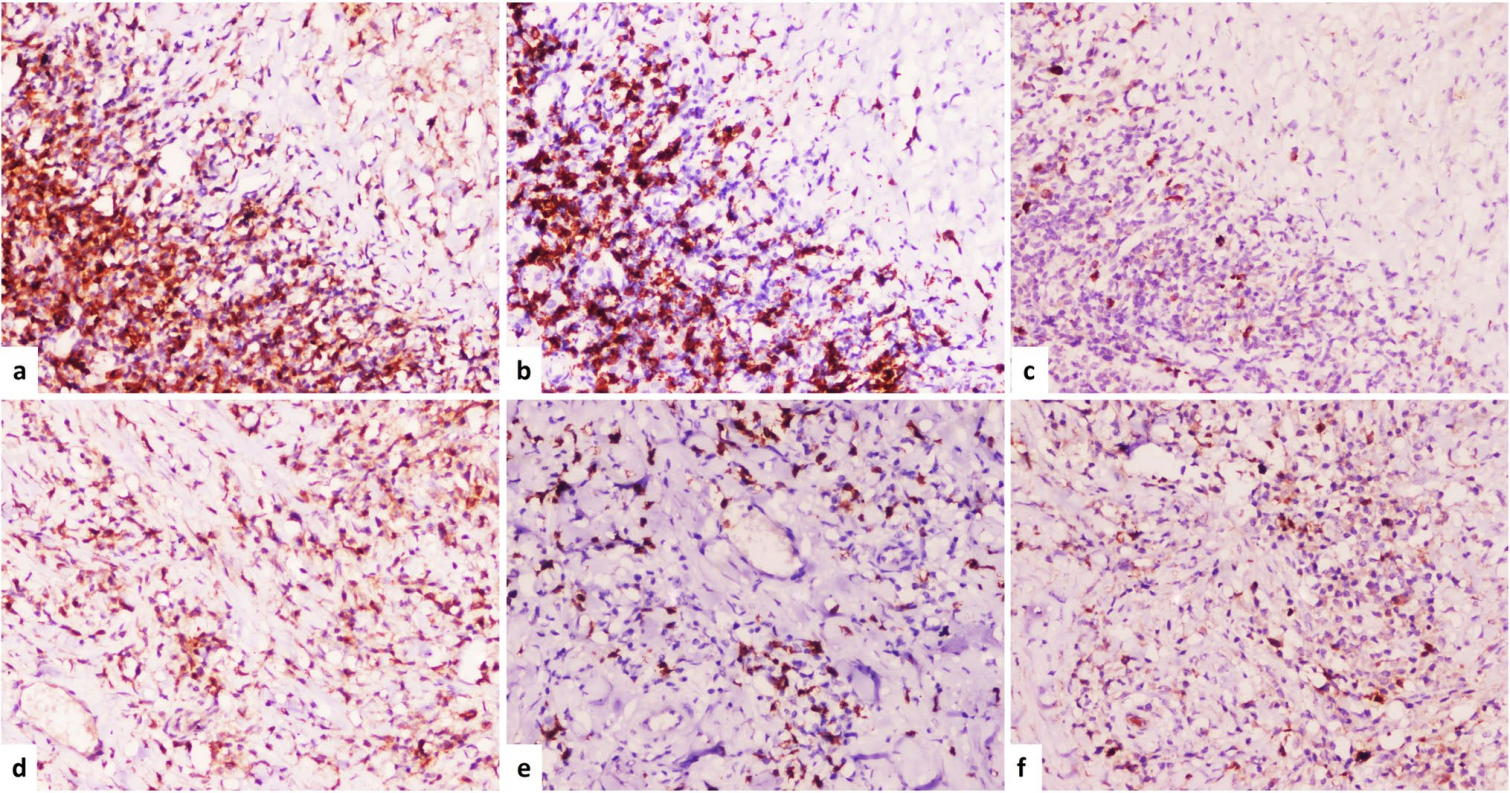
The immunohistochemical expression of CD4, CD8, and GrB in fertile and non-fertile hydatid cysts. **a** The expression of CD4 + T cells in fertile hydatid cysts. **b** The expression of CD8 + T cells in fertile hydatid cysts. **c** The expression of GrB positive cells in fertile hydatid cyst. **d** The expression of CD4 + T cells in non-fertile hydatid cysts. **e** The expression of CD8 + T cells in non-fertile hydatid cysts. **f** The expression of GrB positive cells in non-fertile hydatid cyst, (IHC, × 100, for all)

The fertile and non-fertile groups shared similar CD4 + and CD8 + T cell levels. The non-fertile/non-viable group showed a relatively low level of CD4 + , while CD8 + T cells showed the same value as the other groups. In addition, the three groups showed a high CD4 + /CD8 + T cells ratio. The GrB expression was significantly observed in the non-fertile groups compared to the fertile group (*p* = 0.041) (Fig. 3).

Detailed markers expression is illustrated in Table 3.

**Table 3:** Comparison between different hydatid cysts regarding CD4, CD8, and granzyme B immunohistochemical expression

Variables	Fertile	Non-fertile/viable	Non-fertile/non-viable	*p*-value test
CD4% (mean ± SE)	21.28 ± 4.09	23.67 ± 3.92	8.60 ± 3.82	*p* = 0.34
CD8% (mean ± SE)	8.38 ± 1.02	11.38 ± 1.75	9.60 ± 4.57	*p* = 0.32
CD4 + /CD8 +	2.62 ± 0.35	2.55 ± 0.37	1.21 ± 0.48	*p* = 0.28
CD4 + /CD8 + ratio				
High	22	18	2	*p* = 0.205
Equal	5	2	1	
Low	5	1	2	
GrB				
Positive	17	18	3	*p* = 0.041
Negative	15	3	2	
GrB % (mean ± SE)	0.53 ± 0.09	0.86 ± 0.07	0.60 ± 0.24	*p* = 0.04

GrB expression was significantly associated with the inflammatory density (*p* = 0.005), although it was unaffected by the fibrous deposition within the hydatid cyst wall (*p* = 0.85). Furthermore, non-fertile cysts showed significant GrB overexpression than fertile ones (*p* = 0.04).

### Correlation between CD4 + , CD8 + T cells, and GrB immunohistochemical markers

**Fig. 4:**
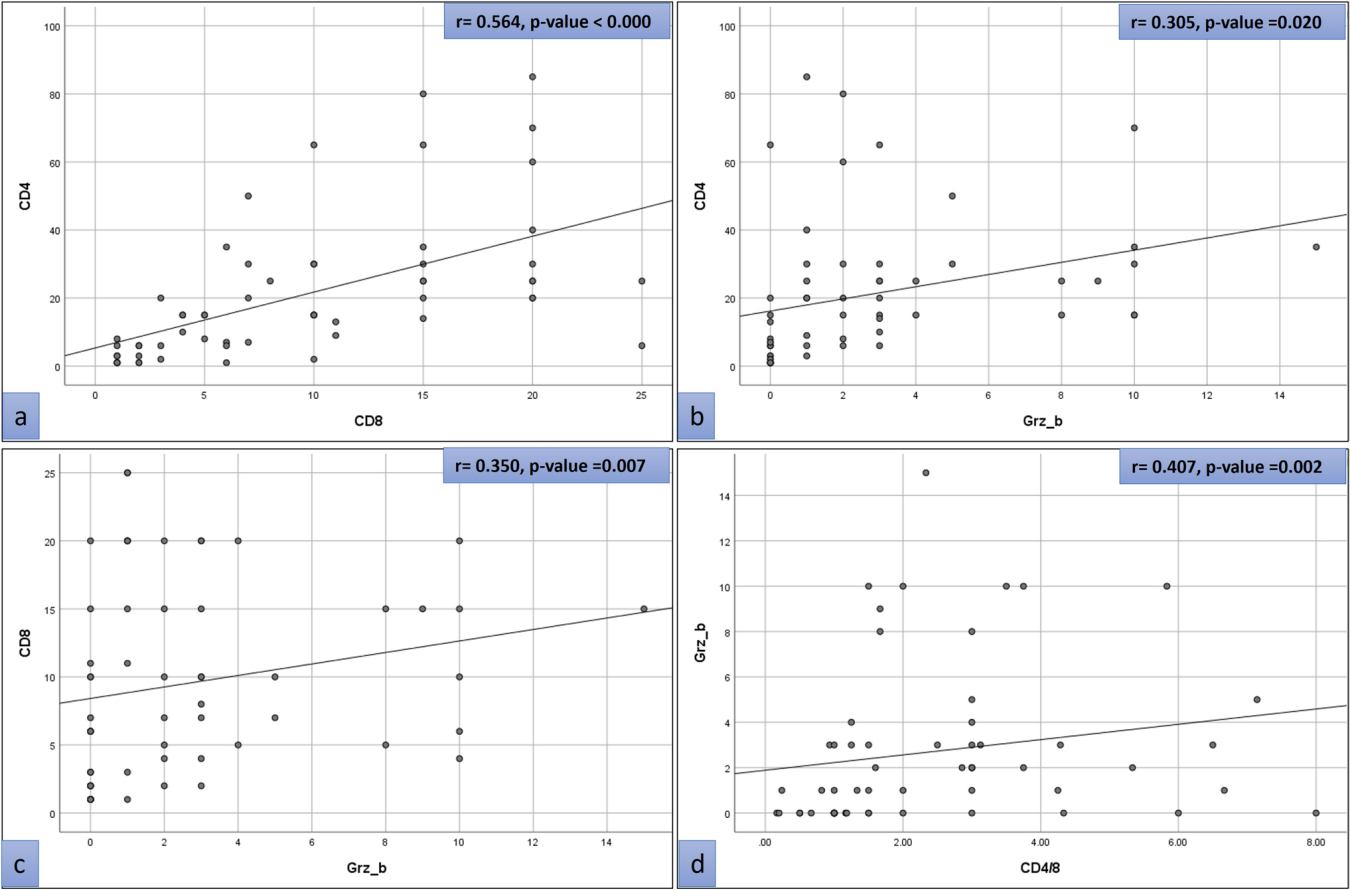
A scatter plot showing the correlation of CD4 + , CD8 + count, and GrB results in hydatid cyst. **a** There is a positive linear correlation between the expression of CD4 and CD8 (*r* = 0.564, *p* < 0.000). **b** There is a positive linear correlation between the expression of CD4 and GrB (*r* = 0.305, *p* = 0.020). **c** There is a positive linear correlation between the expression of CD8 and GrB (*r* = 0.350, *p* = 0.007). **d** There is a positive linear correlation between the expression of GrB and CD4 + /CD8 + ratio (*r* = 0.407, *p* = 0.002)

The study revealed a significant positive association between CD4 + and CD8 + T cells, exhibiting a correlation coefficient of 0.564 with a *p*-value < 0.001. Moreover, a significant correlation was observed between GrB expression and CD4 + and CD8 + T cells, as indicated by its positively correlated results with elevated CD4 + /CD8 + T cells, as illustrated in Fig. 4.

## Discussion

Hydatid disease is considered an important zoonotic disease (Zhang et al. 2012). In Egypt, hydatid disease is still an endemic health problem, and the risk factors for its infection and transmission exist (Heikal and El-Lessy 2021). The present study showed a predominant occurrence of hydatid cysts in adults independent of gender prevalence. Previous studies revealed variability of hydatid cyst occurrence based on the geographical region, culture, and workforce (Khazaei et al. 2016). Hydatid cysts can occur in wide age ranges from 20 to 69 years; however, the adult-onset represents the highest proportion of cases (Khazaei et al. 2016). The lack of gender prevalence of hydatid cysts could result from the equal contribution of both males and females in our region in agricultural and animal husbandry. However, different regions showed variable gender distribution, assuming who comes much with contact with soil and animals (Rezaei et al. 2014; Ilbeigi et al. 2015). Others postulated the female predominance to the genetic susceptibly to different autoimmune diseases. The genetic, environmental, and autoimmune background is thought to induce CE infection (Grubor et al. 2017).

In the present study, the serological test was positive in nearly half of the evaluated cases independent of the type of cyst, fertile versus non-fertile. The diagnosis of a hydatid cyst depends mainly on the radiological findings. Serological studies were reported in cases where imaging results were inconclusive. Serology has relatively low sensitivity and specificity, making them complementary diagnostic tools (Santivañez et al. 2012). The absence of local strains for CE detection could result in its limited validity (El-Sherbini et al. 2020).

The present investigation revealed that there was no statistically significant difference between the fertile and non-fertile cysts regarding the clinical and serological data. Fertile cysts have been documented in patients with negative serology; consequently, negative serology should not rule out hydatidosis because early stages are associated with negative serology (Tamarozzi et al. 2021). Khammari et al. found that neither gender nor age seemed to impact cyst fertility (Khammari et al. 2019). In addition, the possibility of fertile and non-fertile cysts could be detected in the same patient. Previous studies found that cyst number and size are not always reliable indicators of fertility (Manterola et al. 2006). In addition, serological tests’ role in detecting the active form of cysts is controversial (Lissandrin et al. 2018). False-negative results are reported in small, intact cysts, extrahepatic locations, or non-viable cysts (Sarkari and Rezaei 2015). The compromised immune response is attributed to the decay of the germinative membrane, and the potential negative outcomes may arise from the calcification of non-fertile cysts (El-Sherbini et al. 2020).

The current study showed no difference in the severity of inflammation in fertile and non-fertile cysts. A possible mechanism is the ability of CE to evade immune response. In addition, the parasite produces highly immunogenic AgB, which could act directly to innate and adaptive immunity and participate in parasitic adaptation metabolically to the host microenvironment. Their existence in the hydatid cyst fluid possibly guarantees parasite survival (Chemale et al. 2005).

The current investigation demonstrated the existence of CD4 + T and CD8 + T lymphocyte cells within various categories of hydatid cysts. The current state of data suggests that T lymphocytes are the primary inflammatory cells present at the location of local host responses to hydatid cysts in both human and livestock populations affected by CE, as per the findings of Jafari et al. (Jafari et al. 2019). In vitro, it was observed that T cells constituted the predominant infiltrating cells in the vicinity of the established hepatic CE lesions (Li et al. 2019).

The early expression of CD4 + T and CD8 + T cells during infection may suggest their regulatory function in the sustenance and proliferation of protoscoleces. The current investigation has identified a positive correlation between CD4 + T and CD8 + T cells, potentially ascribed to the equilibrium between CD4 + T cell-mediated cellular immune responses and IL-10-generated CD8 + T cells [28]. Similarly, CD4 + Th1 cells have a role in activating CD8 + T under the effect of interferon-γ during infection (Li et al. 2019).

The lack of any significant difference between the expression of CD4, CD8, and even the CD4 + /CD8 + ratio and the fertility of cysts in our study could be related to the ability of EC to induce chronic infection in the human liver (Brunetti et al. 2011). Persistent infection triggers an immune dysregulation in the hepatic tissue, leading to the gradual establishment of an immune microenvironment (Jafari et al. 2019; Wen et al. 2019). Moreover, there is a debate regarding the involvement of CD4 + T and CD8 + T cells in advancing hydatid cysts. The dominant cell populations observed at the location of host tissue responses in humans and sheep are CD4 + T-cells (Vatankhah et al. 2015; Vismarra et al. 2015). Furthermore, a gradual dose-dependent increase was observed in the CD4 + /CD8 + T cell ratio during the early stages of infection (Li et al. 2019). On the other hand, Hou et al. have indicated that reducing CD4 + T cells may facilitate the proliferation and maturation of protoscoleces into hydatid cysts. The findings suggest a potential involvement of CD4 + T cells in the suppression and elimination of hydatid cysts (Hou et al. 2022). The CD8 + T cells that undergo local expansion are implicated in suboptimal outcomes during helminth infections and may also be accountable for immune responses that exhibit suppressive properties (Metwali et al. 2006).

Expression of GrB was positively correlated with CD4 + , CD8 + T cells, and high CD4 + /CD8 + T in the current study. Crosstalk between CD4 + and CD8 + T cells and GrB has been reported. GrB is one of the NKs, CD4 + , and CD8 + T cell-related molecules, representing an active component of host innate immune cells (Campos et al. 2020). GrB is expressed primarily by activated memory CD8 + and memory CD4 + T cells and NK cells during infections and inflammation (Lin et al. 2014). Activated CD4 + T cells express GrB and acquire lytic activity similar to CD8 + T cells. Antigen-specific CD4 + T cell lytic activity mediated by GrB can be triggered by viral and bacterial infections (Brown et al. 2009). GrB is required in CD4 + T cell differentiation and contributes to regulatory CD4 + T cells in controlling immune response through induction of cell apoptosis (Hoek et al. 2021).

The significant association of GrB and elevated CD4 + /CD8 + ratio could indicate a possible harmful effect of GrB activation. Overexpression of GrB produced in chronic leishmaniasis was linked to adverse disease immunopathology (Campos et al. 2020). Similarly, GrB-producing CD4 + T cell responses predict the development of severe host immune response, worsening inflammation, and tumor progression (Park et al. 2021). GrB is emerging as a multifunctional pro-inflammatory protease involved in the pathogenicity of inflammatory diseases, either acute or chronic onset (Velotti et al. 2020). GrB has been claimed to induce fibrosis in different human diseases. The activation of GrB is characterized by excessive apoptosis and abnormal extracellular matrix remodeling, which can lead to tissue fibrosis and impair the affected organ's function (Mack 2018; Velotti et al. 2020).

## Conclusions

CD4 + T and CD8 + T cells are essential inflammatory components in hydatid cysts independent of cyst types. GrB expression in hydatid cysts could enhance the inflammatory response and hinder cyst fertility but does not affect the cyst wall fibrous deposition. We recommend conducting more in vitro and genetic research to confirm our findings and assess any potential therapeutic advantages of targeting GrB in the management of hydatid cysts.

## Data Availability

The datasets created and evaluated in the present investigation can be obtained from the corresponding author upon a reasonable request.

## Abbreviations

CE: Cystic echinococcosis
*E. **granulosus*: *Echinococcus* *granulosus*
WHO: World Health Organization
AgB: Antigen B
Th: T-helper
GrB: Granzyme B
NK: Natural killer
HPF: High power field
H&E: Hematoxylin and eosin
MT: Masson trichrome
